# Prior therapies as prognostic factors of overall survival in metastatic castration-resistant prostate cancer patients treated with [^177^Lu]Lu-PSMA-617. A WARMTH multicenter study (the 617 trial)

**DOI:** 10.1007/s00259-020-04797-9

**Published:** 2020-05-08

**Authors:** Hojjat Ahmadzadehfar, Kambiz Rahbar, Richard P. Baum, Robert Seifert, Katharina Kessel, Martin Bögemann, Harshad R Kulkarni, Jingjing Zhang, Carolin Gerke, Rolf Fimmers, Clemens Kratochwil, Hendrik Rathke, Harun Ilhan, Johanna Maffey-Steffan, Mike Sathekge, Levent Kabasakal, Francisco Osvaldo Garcia-Perez, Kalevi Kairemo, Masha Maharaj, Diana Paez, Irene Virgolini

**Affiliations:** 1 Department of Nuclear Medicine, University Hospital Bonn, Bonn, Germany; 2 Department of Nuclear Medicine, Klinikum Westfalen, Am Knappschaftskrankenhaus 1, 44309 Dortmund, Germany; 3 Department of Nuclear Medicine, University Hospital Muenster, Muenster, Germany; 4 Center for Radiomolecular Precision Oncology, Zentralklinik Bad Berka, Bad Berka, Germany; 5 Department of Urology, University Hospital Münster, Muenster, Germany; 6 Institute for Medical Biometry, Informatics and Epidemiology, University of Bonn, Bonn, Germany; 7 Department of Nuclear Medicine, University Hospital Heidelberg, Heidelberg, Germany; 8 Department of Nuclear Medicine, LMU, University Hospital Munich, Munich, Germany; 9 Department of Nuclear Medicine, Medical University Innsbruck, Innsbruck, Austria; 10 Department of Nuclear Medicine, University of Pretoria & Steve Biko Academic Hospital, Pretoria, South Africa; 11 Department of Nuclear Medicine, Istanbul University, Istanbul, Turkey; 12 Department of Nuclear Medicine and Molecular Imaging, Instituto Nacional de Cancerología Mexico City, Mexico City, Mexico; 13 Docrates Cancer Center, Helsinki, Finland; 14 Department of Nuclear Medicine, Imaging and Therapy Centre, Durban, KwaZulu-Natal South Africa; 15 Department of Nuclear Sciences and Applications, Nuclear Medicine and Diagnostic Imaging Section, IAEA, Vienna, Austria

**Keywords:** mCRPC, Radioligand therapy, Lu-PSMA, Chemotherapy

## Abstract

**Introduction:**

The impact of prior therapies, especially chemotherapy, on overall survival (OS) in patients with castration-resistant prostate cancer (CRPC) receiving **[**^177^Lu]Lu-PSMA-617 therapy has been the subject of controversy. Therefore, WARMTH decided to plan a multicenter retrospective analysis (the “617 trial”) to evaluate response rate and OS as well as the impact of prior therapies on OS in more than 300 patients treated with ^177^Lu-PSMA-617.

**Materials and methods:**

The data of 631 metastatic CRPC (mCRPC) patients from 11 different clinics were evaluated. According to the inclusion and exclusion criteria, all patients had to have received at least abiraterone or enzalutamide prior to **[**^177^Lu]Lu-PSMA-617 therapy. The patients were divided into three groups: patients who had received prior chemotherapy, patients who avoided chemotherapy, and patients for whom a chemotherapy was contraindicated.

**Results:**

The analysis included the data of 416 patients, with a median age of 71.9 years. At the time of analysis, 87 patients (20,9%) were still alive. A total of 53.6% of patients had received both abiraterone and enzalutamide; 75.5% and 26.4% had a history of chemotherapy with docetaxel and cabazitaxel, respectively. A total of 20.4% had had Ra-223. The median OS was 11.1 months. Prior chemotherapy, the existence of bone and liver metastases, as well as Eastern Cooperative Oncology Group (ECOG) status, were significant prognosticators of worse overall survival in both univariate and multivariate analyses. Patients without any prior chemotherapy showed a significantly longer OS (14.6 months). The median OS in patients who received one or two lines of chemotherapy with docetaxel or docetaxel followed by cabazitaxel, respectively, was 10.9 months and 8.9 months. There was no difference in OS between patients who had not received chemotherapy and patients for whom chemotherapy was contraindicated. The other prior therapies did not have any significant impact on OS.

**Conclusion:**

In the present multicenter analysis, chemotherapy-naïve mCRPC patients receiving [^177^Lu]Lu-PSMA-617 therapy had a significantly longer OS than patients with a history of chemotherapy. This remained independent in the multivariate analysis besides presence of bone and liver metastases as negative prognosticators for survival, whereas an ECOG of 0–1 is associated with a longer OS.

**Electronic supplementary material:**

The online version of this article (10.1007/s00259-020-04797-9) contains supplementary material, which is available to authorized users.

## Introduction

Prostate-specific membrane antigen (PSMA), also known as folate hydrolase I or glutamate carboxypeptidase II, is a type II, 750-amino acid transmembrane protein (100–120 kDa), anchored in the cell membrane of epithelial prostate cells. PSMA is highly expressed in prostate epithelial cells and strongly upregulated in prostate cancer. PSMA expression levels are directly correlated to androgen independence, metastasis, and prostate cancer progression [1]. Nevertheless, PSMA is not specific to prostate cells and is expressed in other normal (e.g., salivary glands, duodenal mucosa, a subset of proximal renal tubular cells, and a subpopulation of neuroendocrine cells in the colonic crypts) and neoplastic (e.g., subtypes of breast cancer, renal cell carcinoma, hepatocellular carcinoma, colon carcinoma, and peritumoral and endotumoral endothelial cells of neovasculature) tissues [2, 3]. PSMA undergoes constitutive internalization and, as such, can serve not only as an imaging biomarker but also as a target for radioligand therapy (RLT) [4]. Thus, PSMA appears to be an appealing molecular target for theranostics in metastatic prostate cancer [5].

In the first patient cohort of ten patients, minimal early side effects and a considerable rate of prostate-specific antigen (PSA) response after one cycle of RLT with **[**^177^Lu]Lu-PSMA-617 (^177^Lu-PSMA-617) was demonstrated [6]. Meanwhile, several retrospective and a few phase 2 prospective studies with a limited number of patients have confirmed the efficacy and low toxicity profile of Lu-PSMA therapy in patients with *metastatic castration-resistant prostate cancer* (mCRPC) [7–12]. According to the retrospective analyses, it seems that Lu-PSMA therapy prolongs overall survival (OS) at least in patients with a positive response to this therapy [12–15]. The impact of prior therapies on the overall survival of these patients has not been straightforward in different publications [12, 13, 16].

All of the studies performed so far suffer from a limited number of patients and heterogeneity regarding prior therapies. Therefore, WARMTH (World Association of Radiopharmaceutical and Molecular Therapy) decided to plan a multicenter retrospective analysis (the “617 trial”) to evaluate response rate and OS as well as the impact of prior therapies on OS in more than 300 patients treated with [^177^Lu]Lu-PSMA-617.

## Materials and methods

### Study population

The study populations targeted in this study were mCRPC patients who underwent radioligand therapy with ^177^Lu-PSMA-617 with at least a 6-month follow-up from the time of the first cycle or who died within this time period.

### Study design

This retrospective, multicentric study assessed response and OS and its prognostic factors in mCRPC patients who underwent ^177^Lu-PSMA-617 therapy according to the inclusion and exclusion criteria of this study (Table 1). The planned number of included patients for the analysis was at least 300 patients.

**Table 1:** Inclusion and exclusion criteria

Inclusion criteria	
Castration-resistant metastatic prostate cancer	
Age > 18 years	
RLT using 177Lu-PSMA-617	
Documented progressive disease prior to the first cycle according to PSA and/or imaging	
History of therapy with abiraterone or enzalutamide or both, or documented progressive disease under ongoing therapy with one of these agents (prior to 177Lu-PSMA)	
PSMA-positive metastases (in PSMA scan)	
ECOG 0–2	
GFR > 40 mg/dl	
Have a follow-up at least 6 months from the time of the first cycle, or the patient died within this time	
Exclusion criteria	
Hormone-sensitive prostate cancer	
Active malignancy other than prostate cancer	

### Methodology

First, we designed an Excel table as well as case report forms (CRF) for collecting the data. The centers were free to choose between the Excel sheet or CRF form. The anonymized data were sent to the Department of Nuclear Medicine, University Hospital Bonn, for analysis. This retrospective study was approved by the Ethical Committee of the Medical University of Innsbruck. Radiolabeled peptides were used according to the updated Declaration of Helsinki. All local regulations were observed in the participating countries. Informed consent for performing the therapy as compassionate use, according to local laws in participating countries, were obtained from each patient prior to the administration of radiopharmaceuticals. Due to the retrospective design of the study a formal consent to participate in the study was waived according to local regulations.

### Inclusion criteria

The inclusion and exclusion criteria of this study are shown in Table 1. Only patients who were treated with ^177^Lu-PSMA-617were included in this study. Primary inclusion criteria to be considered for ^177^Lu-PSMA-617RLT was a PSMA-positive scan in patients with progressive disease (PSA and/or imaging). All of the patients had to have been pretreated with abiraterone or enzalutamide or both, or documented progressive disease under ongoing therapy with one of these agents. Prior chemotherapy was not part of the inclusion criteria, because in routine practice, some patients avoid getting chemotherapy, or for some, chemotherapy is contraindicated. To track the effects of chemotherapy as a prior treatment, we divided the patients into three groups: those without prior chemotherapy because of avoidance, those without chemotherapy because of contraindications, and those with a history of prior chemotherapy.

### Response

Changes in the PSA level were classified as either a decrease of ≥ 50% or any percentage decrease in PSA (any PSA decline). Any increase in PSA was considered to indicate disease progression. According to our previous studies [13, 14, 17–19], responders to the first cycle of Lu-PSMA tend to live significantly longer than non-responders. Because of this fact and due to homogeneity in the group of patients regarding response, only the response to the first treatment cycle was considered as a possible predictive parameter in this study.

### Overall survival

Overall survival was defined as the time from the date of the first ^177^Lu-PSMA-617 treatment until death from any cause or until the last follow-up.

### Possible prognostic factors for overall survival

The following pre-therapeutic parameters were evaluated: age; Gleason score; prior therapies including abiraterone, enzalutamide, and first- and second-line chemotherapy; the existence of bone, lymph node, liver, and lung metastases; and the Eastern Cooperative Oncology Group (ECOG) score.

### Statistical analysis

Kaplan-Meier estimators were used to compare survival times between different subgroups of the study population. *p* values were derived from the log-rank test. Uni- and multifactorial hazard ratios were estimated by fitting respective Cox-regression models to the data. *p* values in the scope of the multivariant analyses are Wald *p* values for the respective parameter estimates. All analyses were done with SAS 9.4 (SAS Institute Inc., Cary, NC, USA).

## Results

### Patients

The data of 631 patients treated between February 2014 and December 2018 in 11 centers were collected. The list of the participating sites is shown in accessory Table 1. After the first analysis of the data, 215 patients had to be excluded from the main analysis. The main reason for this exclusion was unclear and incomplete follow-up data.

Four-hundred sixteen mCRPC patients with a median age of 71.9 years (range 43–90) were included. The patients received a total of 1493 cycles (1–12 cycles) of therapy with ^177^Lu-PSMA-617. In median, 3 cycles were applied. The median Gleason score (GS) was 8 (4–10). The mean and median baseline PSA value were 580 and 177 ng/ml, respectively. The age of the patients at the time of diagnosis was documented in 362 patients. The median time between the initial diagnosis and ^177^Lu-PSMA-617 therapy was 7.1 years (range 1–31 years). The majority of patients suffered from bone metastases (92.8%), and 20.9% of patients had liver metastases (Table 2).

**Table 2:** Prior therapies

Parameter		
*n* of patients	416	
Age (mean)	71.9 (range 43–90)	
Gleason score^1^	≤ 7	> 7
	114 (27.4%)	239 (57.5%)
Baseline PSA (ng/ml) mean; median; (range)	580;177; (0.4–11,830)	
Prior therapies	Hx of *n* (%)	Ongoing *n* (%)
Abiraterone^2^	246 (59.1)	76 (18.3)
Enzalutamide^2^	200 (48.1)	114 (27.4)
Docetaxel	314 (75.5)	
Cabazitaxel	110 (26.4)	
Ra-223	85 (20.4)	
ECOG^3^	*n* of patients (%)	
0	156 (37.5)	
1	166 (39.9)	
2	72 (17.3)	
Number of cycles	Sum; median; (range) 1493; 3; (1–12)	
Extent of disease	*n* of patients (%)	
Bone metastases	386 (92.8)	
Lymph node metastases	329 (79.1)	
Liver metastases	87 (20.9)	
Lung metastases	68 (16.3)	
Brain metastases	10 (2.4)	

^1^GS of 63 patients was unknown

^2^223 patients (53.6%) received both abiraterone and enzalutamide

^3^ECOG of 22 patients (5.3%) was not reported

At the time of this analysis, 87 patients were still alive (20.9%). The majority of the patients presented with an ECOG of 0 or 1 (77.4%) (Table 2).

### Prior therapies

Table 2 shows the prior therapies. According to the inclusion criteria, all patients had to have been treated with at least abiraterone or enzalutamide and had documented failure of therapy with these agents. The reason that some patients took abiraterone or enzalutamide concurrently despite progressive disease under these agents was that their urologist or oncologists did not want to stop these medications because clinical benefit was still assumed. Two hundred twenty-three patients (53.6%) had taken both abiraterone and enzalutamide. A first-line chemotherapy with docetaxel had been applied in 314 patients (75.5%).

Of the patients who did not get first-line chemotherapy, chemotherapy had only been contraindicated in 18.8%. Prior to RLT, second-line chemotherapy with cabazitaxel was given in 110 patients (26.4%). Second-line chemotherapy was contraindicated in 13.2% of patients (Table 2).

A prior treatment with radium-223 was given in 85 patients (20.4%). The median interval between the last application of radium-223 and the first cycle of ^177^Lu-PSMA-617 therapy was 3.9 months (range 1–36.4 months).

### Response to the first cycle measured by PSA

PSA values, which were measured 2 months after the first cycle, were available in 393 patients. Two hundred eighty-two (71.8%) patients showed PSA decline, of whom 163 patients (41.5%) showed a PSA decline of ≥ 50%. One hundred eleven patients (28.2%) showed an increase in PSA.

### Overall survival

The median OS was 11.1 months (95% CI 9.7–12.5 months). Table 3 shows the prognostic value of different pre-therapeutic parameters regarding OS in detail. Prior chemotherapy, the existence of bone and liver metastases, as well as ECOG status, was significant prognosticators of worse overall survival in both univariate and multivariate analyses.

**Table 3:** Prognostic value of different pre-therapeutic parameters regarding OS

			Univariate analysis		Multivariate analysis	
Parameter	Patients (*n*)	mOS^$^ (months) (95% CI)	HR (95% CI)	*p* value	HR (95% CI)	*p* value
Age (years)						
≤ 70 > 70	185 231	11.302 (9.205–13.399) 11.105 (9.719–12.490)	1.010 (0.812–1.256) 1 (reference)	0.931		
Gleason score						
≤ 7 > 7	114 239	11.598 (9.503–13.692) 10.415 (8.645–12.185)	1 (reference) 1.325 (0.912–1.924)	0.132		
Abiraterone						
Hx^1^ of Ongoing No	246 76 94	11.598 (9.243–13.952) 10.908 (9.123–12.692) 11.039 (8.582–13.496)	0.913 (0.689–1.212) 1 (reference) 0.869 (0.621–1.216)	0.420		
Enzalutamide						
Hx of Ongoing No	92 48 58	12.287 (10.459–14.116) 10.776 (8.563–12.989) 11.302 (8.473–14.131)	0.772 (0.598–0.997) 1 (reference) 0.859 (0.641–1.152)	0.771*		
Abi/Enza^2^						
Both Only one of them	223 193	11.269 (9.115–13.423) 11.105 (9.220–12.990)	1.064 (0.856–1.323) 1 (reference)	0.574		
First-line CTx^3^						
Hx of No	314 102	10.316 (0.797–8.755) 14.587 (10.331–18.844)	1.495(1.149–1.946) 1 (reference)	0.003	1.557(1.196–2.029)	0.001
First- and second-line CTx						
Hx of only first-line Hx of both No chemotherapy	204 110 102	10.908 (9.050–12.765) 8.936 (6.925–10.948) 14.587 (10.331–18.844)	1.440 (1.088–1.908) 1.679 (1.232–2.288) 1 (reference)	0.01^+^ 0.001	1.495 (1.109–2.017) 1.473 (1.058–2.052)	0.008 0.02
Ra-223						
Hx of No	85 331	10.809 (9.544–12.994) 11.269 (9.544–12.994)	1.132 (0.873–1.469) 1 (reference)	0.354		
ECOG						
0 1 2	156 166 72	16.920 (13.921–19.919) 9.692 (7.460–11.924) 6.341 (9.788–12.619)	0.323 (0.236–0.441) 0.553 (0.412–0.743) 1 (reference)	< 0.0001	0.332 (0.127–0.461) 0.568 (0.421–0.766)	< 0.0001 0.0002
Bone metastases						
Yes No	386 30	10.776 (9.243–12.309) 25.462	2.974 (1.629–5.428) 1 (reference)	0.0004	3.703 (1.900–7.214)	0.0001
Liver metastases						
Yes No	87 329	6.045 (4.744–7.346) 12.977 (11.306–14.649)	2.506 (1.944–3.231) 1 (reference)	< 0.0001	2.394 (1.818–3.153)	< 0.0001
Lung metastases						
Yes No	68 348	11.006 (9.342–12.670) 11.269 (9.613–12.925)	1.048 (0.791–1.390) 1 (reference)	0.743		
Lymph node metastases						
Yes No	329 87	11.203 (9.608–12.798) 11.039 (8.685–13.393)	0.863 (0.665–1.121) 1 (reference)	0.275		

^$^Median overall survival

*There was a significant difference between history of enzalutamide and ongoing usage of enzalutamide (*p* 0.045)

^+^There was no significant difference between patients with only first-line chemotherapy and both chemotherapies

^1^Hx: history

^2^Both abiraterone and enzalutamide

^3^CTx: chemotherapy

**Fig. 1:**
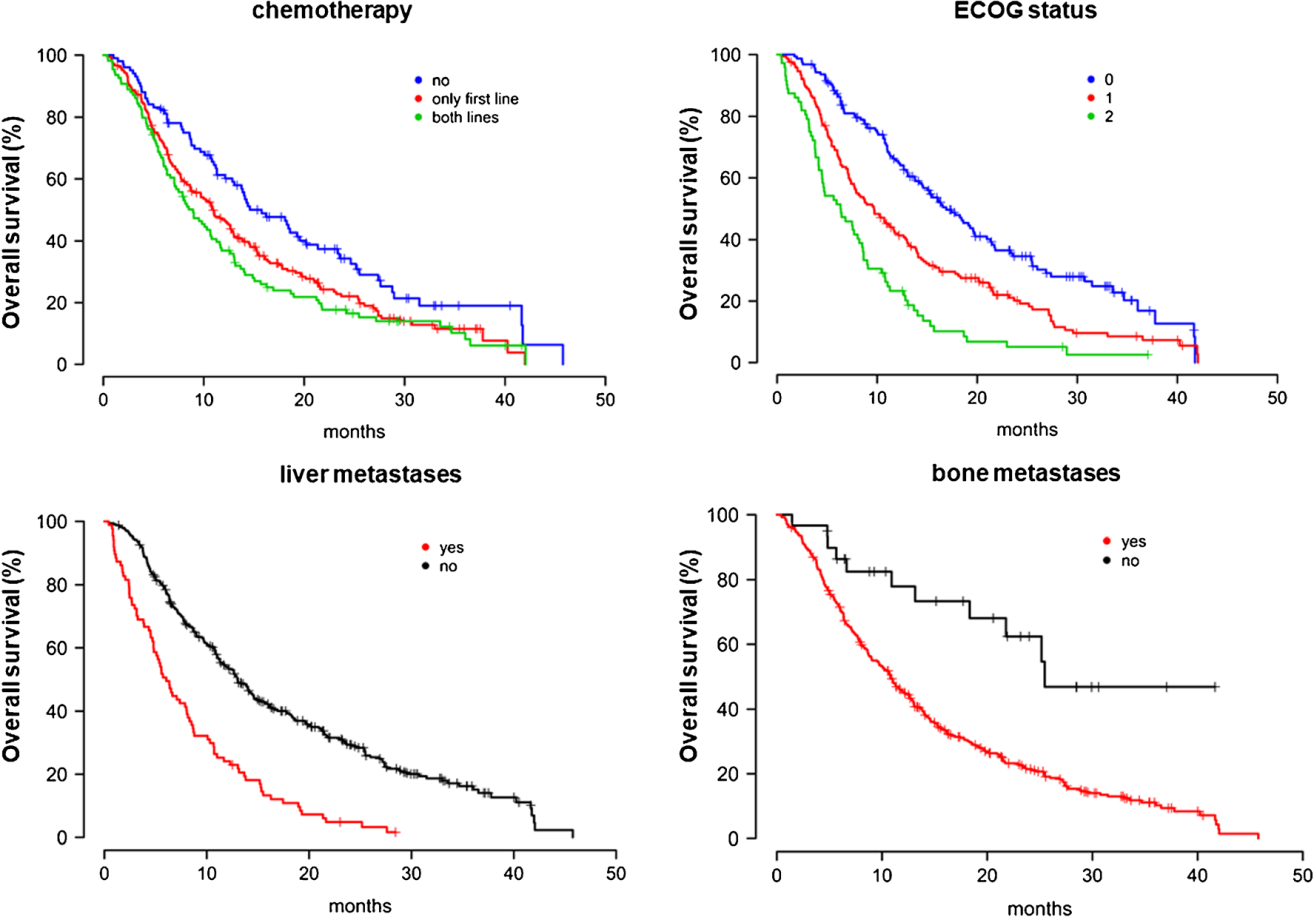
Overall survival of patients for chemotheraphy, liver metastases, ECOG status, and bone metastases

The median OS in patients who received one or two lines of chemotherapy with docetaxel or docetaxel followed by cabazitaxel, respectively, was 10.9 months (95% CI 9.05–12.76) and 8.9 months (95% CI 6.9–10.9). The OS was significantly shorter than in patients without any prior chemotherapy with a median OS of 14.6 months (95% CI 10.3–18.4) (Fig. 1). Out of 102 patients without chemotherapy, 83 patients avoided chemotherapy despite lacking contraindications, and for 19, it was contraindicated. The median OS in the first group was 15.8 months (95% CI 11.4–20.1) and in the second group was 14.0 months (95% CI 3.7–24.4), *p* = 0.29.

Thirty patients without bone metastases (Table 3; supp. Fig. 1) only had lymph node metastases, and these patients showed the longest median OS.

**Fig. 2:**
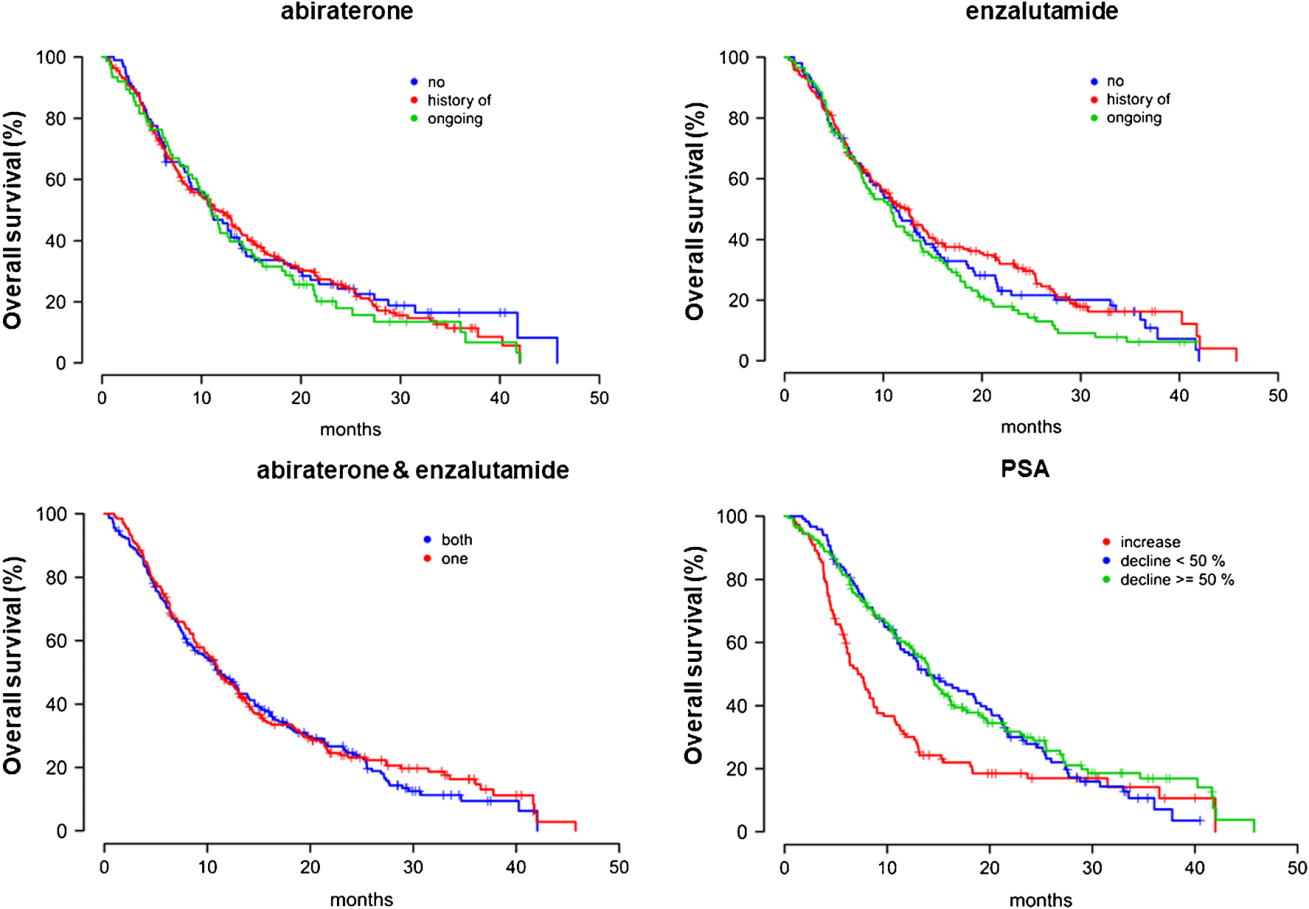
Overall survival of patients who undergone abiraterone, enzalutamide, abiraterone and enzalutamide, and PSA medications

Although a prior anti-hormonal therapy with either abiraterone or enzalutamide or both was not a significant predictive factor, there was a significant difference between the OS of patients with a history of enzalutamide and patients who were under concurrent usage of enzalutamide during ^177^Lu-PSMA-617 treatment (12.3 vs 10.8 months, respectively; *p* = 0.045) (Fig. 2).

**Fig. 3:**
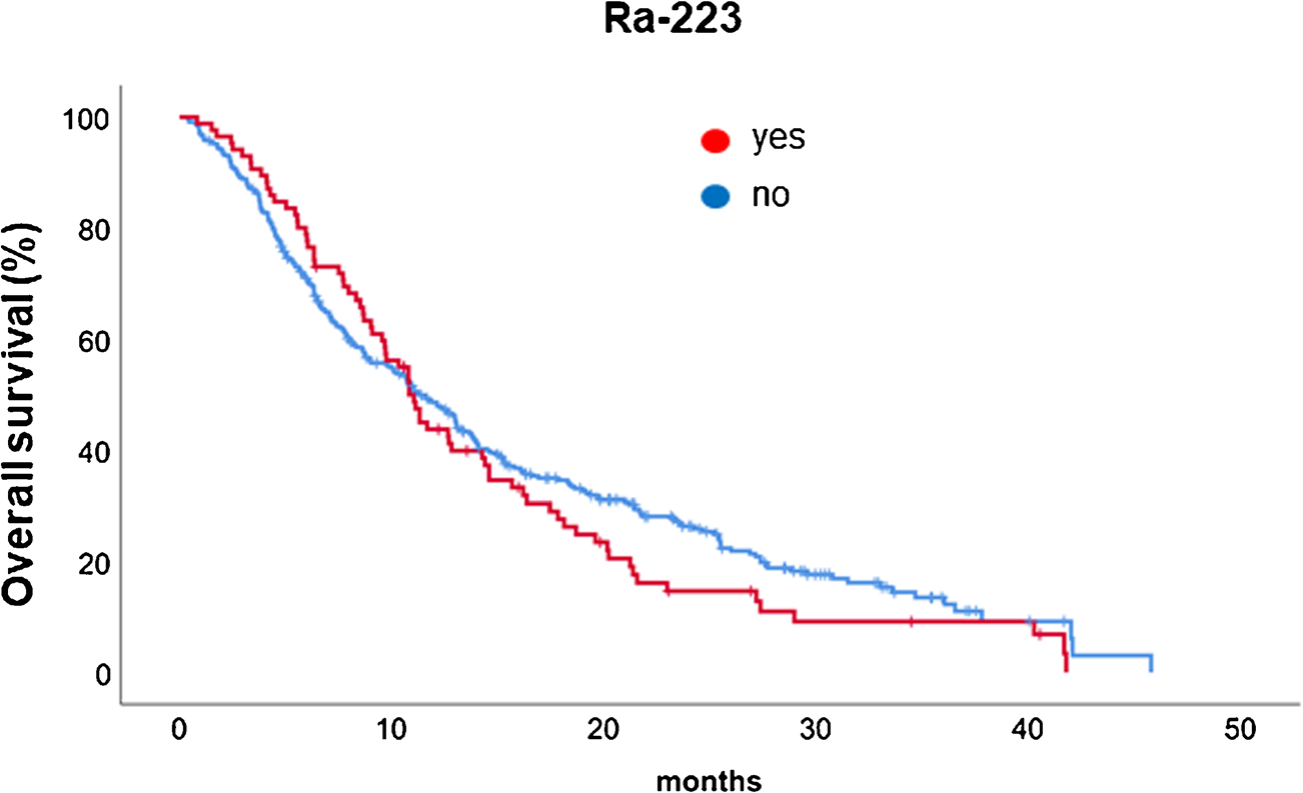
Overall survival of patients who undegone Ra-223 treatment

Patients with prior radium-223 therapy showed a median OS of 10.8 months (95% CI 9.8–11.9 months) vs a median of OS of 11.3 months (95% CI 9.5–13.0 months) in patients without prior radium-223 (*p* = 0.34) (Fig. 3).

The median OS of patients with a PSA decline of less than 50% as well as with a decline ≥ 50% was significantly longer than patients with a rising PSA after the first cycle. The median OS of patients with rising PSA, decline < 50%, and decline ≥50% was 7.2 months (95% CI 5.6–8.7), 13.9 months (95% CI 10.1–17.7), and 14.3 months (95% CI 12.6–15.9), respectively (*p* < 0.0001). There was no significant difference between patients with more or less than 50% PSA decline regarding OS (*p* = 0.6) (Fig. 2).

## Discussion

The approved therapies for patients with mCRPC that can significantly improve OS are next-generation anti-hormonal therapies (abiraterone and enzalutamide), first- and second-line chemotherapies with docetaxel and cabazitaxel, and alpha radionuclide therapy with radium-223 [20]. Currently, several novel agents, such as immunotherapeutics or therapies targeting poly (adenosine diphosphate–ribose) polymerase (PARP) inhibitors, are under advanced clinical investigation [20]. Radionuclide imaging and therapy using PSMA as a target have been investigated for more than two decades beginning with monoclonal antibodies that were later replaced by low molecular weight PSMA ligand inhibitors [21]. Since 2015, there have been increasing numbers of published data showing promising efficacy and a low toxicity profile of these agents in mCRPC patients [6, 7, 11, 22].

Although this therapy is still experimental and is mainly given in compassionate-use scenarios only, different societies have published guidelines or recommendations trying to standardize this therapy for patients with mCRPC [23–26]. The most important limitations of the published data are the limited number of included patients and the heterogeneity of patients regarding prior therapies.

Ahmadzadehfar et al. reported a median OS of 60 weeks in 100 patients who received a median of 3 cycles of ^177^Lu-PSMA-617 (total 347 cycles). All of the patients had a history of therapy with either enzalutamide or abiraterone, or both. At least one line of chemotherapy had been performed in 70% of the patients, and 36% had a history of radionuclide therapy with radium-223. Here, a PSA decline after the first RLT, as well as a decline ≥ 50%, was significant prognosticators of longer OS. Rahbar et al. [14] reported an OS of 56 weeks in 104 patients treated with 351 cycles of ^177^Lu-PSMA-617. All of these patients had a history of therapy with at least one line of chemotherapy, as well as either abiraterone or enzalutamide. Both studies showed that patients who respond to PSMA therapy live longer than those who do not. In these studies, prior therapies, such as chemotherapy, had no impact on OS. Heck et al. [11] reported the results of therapies using [^177^Lu]Lu-PSMA-I&T in 100 patients with mCRPC who received a total number of 319 cycles (median 2 cycles). Here, the median OS was 12.9 months (95% CI 9.9–15.9). The included patients were comparable with those of our cohort regarding prior therapies.

Heck et al. also showed a significant correlation between PSA response under RLT and survival. They analyzed the PSA changes within 12 weeks of RLT. According to their analysis, a maximum PSA decline of ≥ 50% was associated with longer OS (median 16.7 (*n* = 32) vs 6.2 (*n* = 60) months, *p* = 0.007). In our current study, the median OS of patients with rising PSA, decline < 50%, and decline ≥50% were 7.2 months, 13.9 months, and 14.3 months, respectively (*p* < 0.0001); however, there was no significant difference between patients with more or less than 50% decline regarding OS (*p* = 0.6) by measuring PSA 8 weeks after the first cycle. This difference may be due to the measuring time point of PSA. The best response, within 12 weeks, means that the majority of patients got two cycles of therapy; on the other hand, it seems that Heck et al. did not differentiate between rising PSA and a decline of less than 50%.

Despite PCWG3’s suggestion to measure PSA decline after 12 weeks in clinical trials, prior studies as well as the present study found that a good response to the first cycle (a PSA decline, measured 2 months after the first cycle) is associated with a favorable response to further cycles in more than 90% of patients [13, 27].

Barber et al. reported the results of 167 mCRPC patients who were treated with ^177^Lu-PSMA-617 or ^177^Lu-PSMA-I&T [12]. The patients were divided into two groups according to prior therapy with taxane-based chemotherapy. The median OS in 83 patients in the taxane-pretreated group was 10.7 months; it was 27.1 months in 84 patients in the taxane-naïve group [12]. In the taxane-pretreated group, 76% and 14% of patients prior to RLT had received abiraterone or enzalutamide and Ra-223 treatments, respectively, while only 38% and 2% of patients in the taxane-naïve group had received abiraterone or enzalutamide and Ra-223 therapies, respectively [12]. This likely means that the long OS of 27.1 months in the taxane-naïve group was caused by the fact that about 60% of the patients in this group had received an RLT as a first-line therapy, thus the natural course of their prostate cancer was associated with a significantly longer OS than that of the patients who had been treated with various mCRPC-approved compounds prior to ^177^Lu-PSMA-RLT. Thus, despite longer OS in the taxane-naïve group, in the multivariate analysis, prior chemotherapy was not a significant prognosticator of overall survival [12].

In our multicenter analysis, all of the patients had received abiraterone or enzalutamide, and 53.6% had been treated with both agents. A total of 75.5% had at least a history of first-line chemotherapy with docetaxel. A therapy with Ra-223 had been done in 20.4% of the patients. Altogether, the included patients in this retrospective multicenter analysis were heavily pretreated. Despite prior therapies and advanced disease (92.8% and 20.9% bone and liver involvement, respectively), the median OS was 11.1 months (95% CI 9.7–12.5 months) and was comparable with the taxane-pretreated group of Barber et al. and other prior studies [11–14].

In terms of treatment planning, having predictive parameters for a favorable response and prognosticators of longer OS is of importance for us as clinicians, first, to decide on the indication of a therapy, and second, to accurately inform patients about the treatment response rate and their prognosis for survival. In the current study, age, GS, prior therapies with abiraterone or enzalutamide as well as Ra-223, and the existence of lymph node as well as lung metastases were not significant prognosticators of OS, while prior chemotherapy, ECOG status and the existence of bone and liver metastases were prognosticators of OS in both univariate and multivariate analysis.

As mentioned above, a prior therapy with enzalutamide did not have a significant impact on OS; however, there was a significant difference between the OS of patients with a history of enzalutamide and the OS of patients who were under ongoing usage of enzalutamide (12.3 vs 10.8 months, respectively; *p* = 0.045). These findings should be further explored in future studies. One explanation could be the negative impact of enzalutamide on PSMA expression, reducing the tumor-absorbed dose, or an agonistic effect of enzalutamide on patients who do not respond any more to this agent. The potential impact of enzalutamide and abiraterone on the efficacy of PSMA-RLT when used concurrently is therefore very interesting, even more so since the current phase III registrational trial, VISION, tests PSMA-RLT in combination with enzalutamide or abiraterone only. Thus, in the future, depending on the results of VISION [28] and subgroup analyses, a PSMA monotherapy may have to be compared with a combination therapy including abiraterone or enzalutamide. For some patients, PSMA-RLT alone may be sufficient.

Patients who had received a prior therapy of Ra-223 showed a longer OS during the first 10 months as compared with patients without any Ra-223 (Fig. 3). Ra-223 is a bone-seeking alpha-emitting radionuclide acting in reactive bone forming cells adjacent to cancer cells. This therapy has demonstrated a median OS of 12.4 months alone in 150 patients [29]. The early improvement of OS is thus possible due to existing long-acting synergistic Ra-223 effect; this effect lasts typically 4–5 months, and thus an average of 7–9 months benefit is expected. After 10 months, the ^177^Lu-PSMA-617 showed better OS in those patients who did not receive Ra-223.

The existence of visceral metastases is a negative prognostic factor, as other studies have shown [11, 12, 30]. Although in our multicenter study patients without prior chemotherapy showed a significantly longer OS, we should take into consideration that some of these patients who had initially avoided chemotherapy received it after having progressed on PSMA treatment. Future trials will have to elucidate the ideal position of PSMA-RLT within the ever-growing armamentarium of therapies for patients with mCRPC. This issue cannot be analyzed in a retrospective setting, and it should thus be evaluated in a prospective setting.

Since we included patients from different departments, we did not analyze baseline laboratory parameters such as blood count, alkaline phosphatase, LDH, and different tumor markers other than PSA, first, because different laboratories have different ranges of normal, and second, because apart from blood counts, the other parameters were not checked routinely in all clinics.

### Limitations

One of the most important limitations of this study is its retrospective design; however, we tried to exclude all patients with unclear follow-up or documentation from the analysis. The high proportion of patients who were excluded in this analysis is a major drawback of the analysis and is due to the retrospective design and recorded data mostly in clinical routine and different countries.

Additional limiting factor is the timepoint of calculation of survival rates, especially according to performed chemotherapy, this might represent a lag-time bias. This might be overcome with results of the prospective trials, which are currently running [28, 31].

## Conclusion

In the present multicenter analysis, the median OS was 11.1 months, whereas the patients without prior chemotherapy showed a significantly longer OS, 14.6 months. This remained independent in the multivariate analysis besides presence of bone and liver metastases as negative prognosticators for survival, whereas an ECOG of 0–1 is associated with a longer OS. Results of the phase III VISION trial are eagerly awaited to bring this effective therapy to approval.

## Electronic supplementary material

ESM 1 (PNG 246 kb)

High Resolution (TIF 1249 kb)

ESM 2 (DOCX 13 kb)
