# Supplementary figures and images for: Prior therapies as prognostic factors of overall survival in metastatic castration-resistant prostate cancer patients treated with [177Lu]Lu-PSMA-617. A WARMTH multicenter study (the 617 trial)
Source: Eur J Nucl Med Mol Imaging. 2020 May 8;48(1):113–22. doi: 10.1007/s00259-020-04797-9 (PMC7835179; doi:10.1007/s00259-020-04797-9)

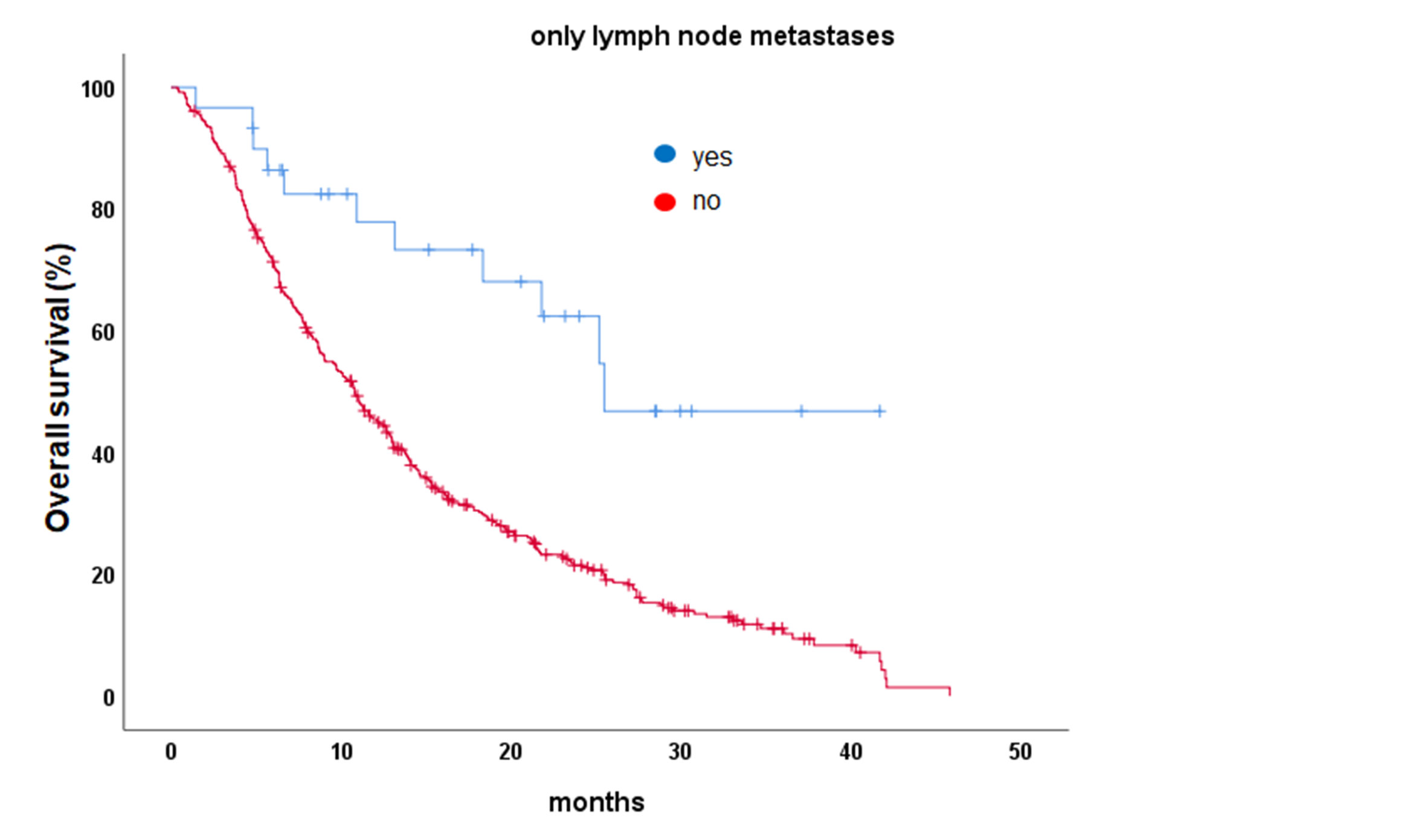

Supplement: Supplementary file 1 — (PNG 246 kb) [file 259_2020_4797_Fig4_ESM.png]

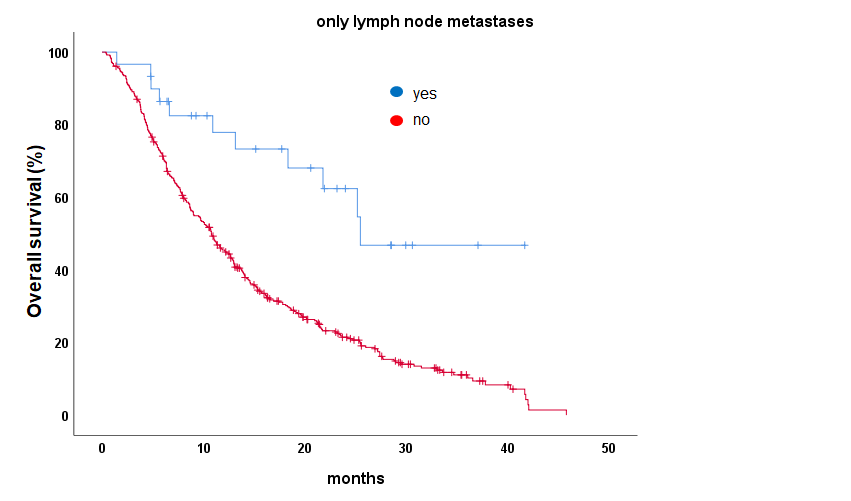

Supplement: Supplementary file 2 — High Resolution (TIF 1249 kb) [file 259_2020_4797_MOESM1_ESM.tif]
