# Supplementary material for: Prior therapies as prognostic factors of overall survival in metastatic castration-resistant prostate cancer patients treated with [177Lu]Lu-PSMA-617. A WARMTH multicenter study (the 617 trial)
Source: Eur J Nucl Med Mol Imaging. 2020 May 8;48(1):113–22. doi: 10.1007/s00259-020-04797-9 (PMC7835179; doi:10.1007/s00259-020-04797-9)
Supplement: Supplementary file 3 — (DOCX 13 kb) [file 259_2020_4797_MOESM2_ESM.docx]

**Accessory table 1**

**The list of participated Departments**

1. Department of Nuclear Medicine, University Hospital Bonn
2. Department of Nuclear Medicine, University Hospital Muenster
3. Center for Precision Radiomolecular Oncology, Bad Berka (ZBB)
4. Department of Nuclear Medicine, University Hospital Heidelberg
5. Department of Nuclear Medicine, LMU, University Hospital Munich
6. Department of Nuclear Medicine, Medical University Innsbruck, Innsbruck, Austria
7. Department of Nuclear Medicine, University of Pretoria & Steve Biko Academic Hospital, South Africa
8. Department of Nuclear Medicine, Istanbul University, Istanbul, Turkey
9. Department of Nuclear Medicine and Molecular Imaging, Instituto Nacional de Cancerología Mexico City, Mexico
10. Docrates Cancer Center, Helsinki, Finland
11. Department of Nuclear Medicine, Imaging and Therapy Centre, Durban, KwaZulu-Natal, South Africa
